# Resistance to cellular HIV infection

**DOI:** 10.1093/emph/eov016

**Published:** 2015-08-21

**Authors:** Alice M. Clomegah, Stephen J. Chapman

**Affiliations:** ^1^ NYU College of Global Public Health New York, NY 10003 USA;; ^2^ Oxford University Hospitals, Oxford OX3 7LE, UK

## GENETIC RESISTANCE TO HIV

**Figure 1.**
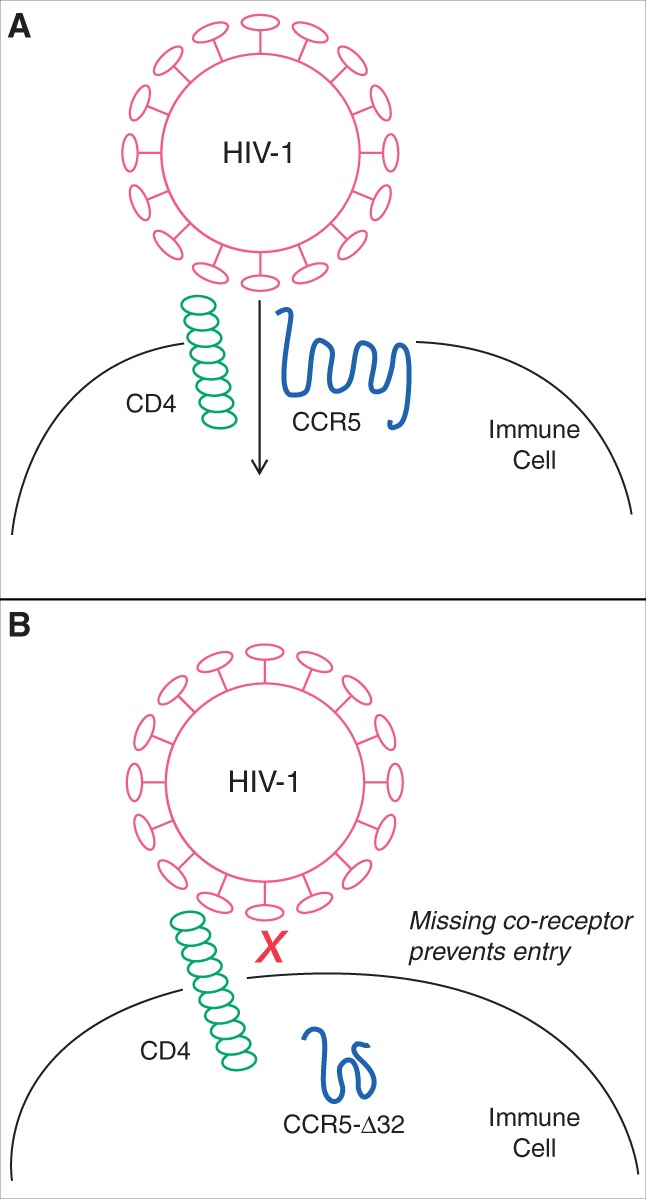
HIV-1 entry into immune cells

Infection with human immunodeficiency virus-1 (HIV-1) remains a major cause of premature death worldwide. In order to enter and infect immune cells, HIV-1 binds to cell surface receptors including the CCR5 chemokine receptor (Fig. 1A). A well-described functional polymorphism in the *CCR5* gene comprises a 32-bp deletion (called *CCR5-Δ32)* which results in a lack of the last three transmembrane domains of the CCR5 protein [1]. The mutated protein is contained in the cytoplasm, resulting in a complete lack of CCR5 cell surface receptor. In the absence of this co-receptor binding site, HIV-1 is unable to enter the cell (Fig. 1B); individuals homozygous for *CCR5-Δ32* display complete resistance to HIV-1, whereas heterozygotes have a delayed onset of AIDS [1].

## EVOLUTIONARY PERSPECTIVES

Chemokines and their receptors play a central role in the trafficking and activation of lymphocytes, but perhaps surprisingly there are no apparent pathological consequences of *CCR5-Δ32* homozygosity. Intriguingly, this variant displays marked population differentiation which would not be explained by the very recent emergence of HIV-1 in sub-Saharan Africa: the *CCR5-Δ32* allele is highly prevalent in Europeans (frequency up to 14%), while very rare or absent among African and Asian populations [2].

Early studies suggested that the *CCR5-Δ32* allele arose relatively recently (∼700 years ago) and was subject to strong positive selection [2]. Infection with *Yersinia pestis*—the cause of the bubonic plague in Europe during this time period—or smallpox were suggested as potential selective factors favoring *CCR5-Δ32* [2]. HIV and smallpox both cause cellular immune dysfunction and both enter leukocytes using chemokine receptors. If exposure to smallpox provided selective pressure favoring *CCR5-Δ32*, it is plausible that exposed populations now have an evolutionary advantage in facing HIV [3]. Recently, however, high-density genetic maps have questioned the evidence for a recent origin of *CCR5-Δ32*, suggesting instead that this allele may have arisen ∼5000 years ago [4], under neutral evolution, or subject to an ancient, currently unknown positive selective pressure.

## FUTURE IMPLICATIONS

Antagonism of CCR5 interferes with HIV-1 cell entry, and indeed the CCR5 antagonist drug maraviroc is approved by the FDA for treatment of exclusively CCR5-tropic viral strains of HIV-1 [5]. The clinical relevance of *CCR5-Δ32* is further illustrated by a case of long-term HIV control following stem-cell transplantation from a *CCR5-Δ32* homozygous donor [6], although viral escape through chemokine receptors other than CCR5 may limit the success of this approach [7]. Of broader relevance, development of a therapy that eliminates the transmembrane portion of the CCR5 protein to mimic the advantage conferred by the natural *CCR5-Δ32* variant might constitute a functional HIV-1 cure. A ‘gene editing’ approach using infusion of autologous CD4 T cells which have been modified by zinc finger nucleases to disrupt *CCR5* (mimicking *CCR5-Δ32*) appears safe and confers partial disease resistance [8].
